# Germline, hematopoietic, mosaic, and somatic variation: interplay between inherited and acquired genetic alterations in disease assessment

**DOI:** 10.1186/s13073-016-0350-8

**Published:** 2016-10-05

**Authors:** Eric Q. Konnick, Colin C. Pritchard

**Affiliations:** Department of Laboratory Medicine, University of Washington Medical Center, Box 357110, 1959 NE Pacific St, Seattle, WA 98195-7110 USA

## Abstract

Advances in genetic analysis have revealed new complexities in the interpretation of genetic variants. Correct assessment of a genetic variant relies on the clinical context and knowledge of the underlying biology. We outline four scenarios encountered in genetic testing where careful consideration of the origin of genetic variation is required for variant interpretation.

## Advances in DNA sequencing

Widespread availability of techniques for deep sequencing of the human genome has accelerated the rate at which the genetic basis of inherited and somatic conditions is revealed. With massively parallel next-generation sequencing, it is possible to accurately detect mutations in small subsets of cells that were undetectable using Sanger sequencing methods. In addition to mutations associated with cancer, low-frequency somatic mutations associated with local tissue proliferations and vascular malformations typical of mosaic overgrowth syndromes have recently been discovered. Extensive genomic analyses of a large number of patients with varying phenotypes have exposed a complex relationship between pathogenic variants identified in the context of inherited and acquired conditions.

## Relationship between genotype and phenotype in inherited and sporadic diseases

Deeper understanding of genotype–phenotype relationships in inherited and somatic disease is enabling innovative clinical diagnostics for precision medicine, but we must understand the range of biological possibilities that may explain the genetic data. For example, classic assumptions about inherited variants associated with cancer predisposition have recently been challenged; these assumptions include the need for multiple affected generations, mutations in specific genes being related to a specific spectrum of cancers, or that variants detected in peripheral blood reflect only the germline. Cancers with a strong gender-specific incidence may be passed through the opposite-sex parental lineage, giving the false impression that there is not a heritable syndrome present [1]. It has also become clear that the genotype–phenotype relationship with disease is broader than previously appreciated [2] and that variants detected in blood may be of somatic origin [3].

**Fig. 1:**
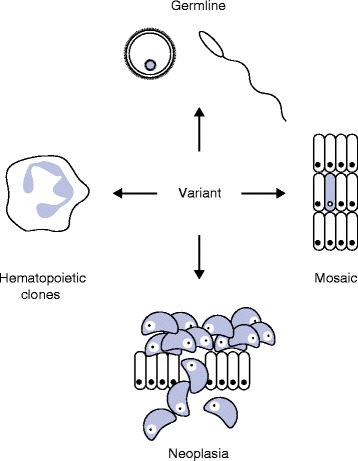
Genetic variation attributable to distinct biological processes. Variants detected by genetic testing may fall into at least four categories, including inherited germline variants, post-zygotic somatic mosaic mutations, lineage-restricted somatic mutations, such as in age-related clonal hematopoiesis, and somatic mutations related to cancer (neoplasia)

Here, we describe four key areas where there is interplay between germline and somatic genetic mutations that need be considered in relationship to an observed phenotype for the correct variant interpretation in a clinical context. The four areas we will address are: 1) germline pathogenic variants discovered as part of tumor-based testing, 2) tumor-based testing performed for the purpose of clarifying germline mutation status, 3) somatic mutations detected in peripheral blood as part of cancer predisposition testing, and 4) mosaic mutations detected in somatic overgrowth syndromes (Fig. 1). Current guidelines for interpretation of inherited genetic variants, such as from the American College of Medical Genetics and Genomics and the Association for Molecular Pathology, do not adequately address clinical context or somatic biological phenomena in the classification schema. Furthermore, there are not yet widely accepted guidelines for interpretation of variants identified in cancer. As the field moves forward, variant interpretation schema that take into account the clinical context of the individual patient, and the biological processes described here, will allow more accurate variant assessment.

## Germline origin for pathogenic variants identified in tumor-based testing

In the course of tumor-based testing, germline cancer predisposition mutations are more frequently identified than in the general population because many cancers have a heritable component. Furthermore, these mutations may not be anticipated because of a lack of a strong family history of cancer, sex-specific incidence of certain neoplasms [1], or incomplete penetrance or hypomorphic mutations [4].

Genomic interrogation of cancers has been undertaken in various forms for decades, but the advent of quantitative, single-nucleotide-resolution data of genetic aberrations in cancer has been revolutionary. Recent data reveal that pathogenic genetic variants identified within cancer tissues are of germline origin in about 10 % of both childhood and adult cancers unselected for family cancer history [5, 6]. In these studies, loss of heterozygosity or additional somatic mutations suggest that germline mutations were significantly related to the development of cancer. These findings highlight that one must consider the possibility of a germline origin for pathogenic variants when evaluating cancer tissue, even in the absence of a family history.

## Variation in genetic mechanisms for a tumor phenotype

Cancer tissue can provide useful information regarding the origin of an observed phenotype and for inferring germline genetic status. The best example of this is Lynch syndrome, a cancer predisposition syndrome caused by inherited mutations in mismatch repair genes. Diagnostic algorithms have been developed to help identify individuals who are at risk of carrying a germline mutation based on the tumor phenotype, and these individuals are typically referred for germline analysis of the implicated genes if the screening test results are abnormal. People with abnormal screening results can often be classified with regard to their germline genetic risk of Lynch syndrome, but a subset of patients cannot be classified using the commonly used methods and are generally treated as carriers of risk alleles that cannot be currently identified (“Lynch-like” or “suspected Lynch”) [7, 8]. Genomic analysis of cancer tissues in this subgroup of patients has revealed that up to 70 % of these unresolved cases are due to multiple somatic mutations in mismatch repair genes that explain the screening results [8]. Identification of somatic mutations as the cause of a positive Lynch-syndrome-screening test allows de-escalation of ongoing cancer-surveillance programs in these individuals and their families. Correct classification of tumors into sporadic or Lynch syndrome-related is important for future surveillance activities or selecting effective therapies, regardless of the genetic origin of their mismatch repair deficiency. This example highlights that genetic mechanisms for a tumor phenotype may be varied, with implications for screening, diagnosis, prognosis, and therapeutic management.

## Somatic alteration of hematopoietic cells

Germline genetic testing for cancer predisposition genes may reveal mutations that are not at the expected heterozygous or homozygous ratios for a germline variant. When this is due to a population of cells that underwent a somatic mutation during development (post-zygotic mosaicism) it is relevant to cancer predisposition, so it is important to identify such individuals when they present for testing. Importantly, recent data suggest that a likely explanation for many of these cases is the detection of hematopoietic cells that have experienced a somatic alteration due to age [3] or chemotherapy exposure [9]. To complicate matters, sensitive screening methods currently in clinical use for non-cancer applications can detect mutations from cancer cells that have shed cell-free DNA in the peripheral blood. Detection of these phenomena will become more commonplace as more individuals undergo evaluation using broad genetic testing panels, in both clinical care and direct-to-consumer settings. These observations reinforce the importance of integration of the genetic test results with the patient phenotype, family history, and other factors when assessing the significance of genetic variants.

## Activating mutations resulting in somatic overgrowth syndromes

Numerous syndromes are now described in which overgrowth of somatic tissues is a part of the spectrum of the condition [10]. Application of sensitive genomic techniques has revealed that the affected tissues in these individuals often contain post-zygotic mosaic somatic alterations that are identical to those observed in cancer, such as *PIK3CA* hotspot mutations. The identification of activating mutations in the setting of somatic overgrowth syndromes not only is important for diagnosis but may also have implications for targeted therapies and requires selection of appropriate tissue sources in addition to the analytical skills needed to arrive at a clinically meaningful result.

## Conclusions

We have briefly outlined four scenarios in which there is overlap between genomic variants relevant to germline, somatic, and invasive neoplastic disease. There is likely to be more complexity that we have yet to uncover and investigations into the contributions of epigenetic regulation, host–microbiome interaction, or other research avenues may reveal further relationships. In the coming decade, as individual genomic analyses across a wide variety of phenotypes become more common, the depth of understanding of the described associations will increase. To appropriately counsel clinicians and patients on the interpretation of genetic testing results, laboratory providers and researchers must appreciate the relationship of an identified genetic variant, the clinical context, and the potential biological processes at play. Incorporation of these factors into professional genetic variant interpretation guidelines in the coming years will help increase recognition of the overlap between genetic variants due to germline, somatic, or hematopoietic processes and harmonize interpretation across a wide spectrum of researchers and providers.
